# Racial and Ethnic Disparities in Community-Based Pharmacies: A Scoping Review

**DOI:** 10.3390/pharmacy11030093

**Published:** 2023-06-02

**Authors:** Tamera D. Hughes, Jessica S. Roller, Faustina Hahn, Stefanie P. Ferreri

**Affiliations:** UNC Eshelman School of Pharmacy, University of North Carolina at Chapel Hill, Chapel Hill, NC 27599, USA

**Keywords:** pharmaceutical care, healthcare disparities, community pharmacy, racial disparities, ethnic disparities, healthcare systems

## Abstract

As pharmacy practice shifts its focus toward population health care needs that serve public health, there is a need to understand community-based pharmacies’ contributions to the reduction in health disparities. A scoping review was conducted to identify what community-based pharmacies in the United States are doing to target racial and ethnic disparities in community-based pharmacies. Forty-two articles revealed that community-based pharmacy services addressed racial and ethnic inequities in a variety of ways, including the types of interventions employed, as well as the ethnicities and conditions of the sample populations. Future work should focus on ensuring interventions are carried out throughout pharmacy practice and accessible to all racial and ethnic minoritized populations.

## 1. Introduction

Racial and ethnic healthcare disparities are prevalent throughout the United States [1]. While this issue has received considerable attention from researchers, health practitioners, academics, and policymakers, disparities between White people and Black, Indigenous, and People of Color (BIPOC) persists—particularly in access to health care, disease incidence and prevalence, and mortality [2,3,4,5,6,7]. Over the past several decades, there have been various calls to change how race and racism are examined in healthcare [8]. In 2003, in *Unequal Treatment: Confronting Racial and Ethnic Disparities in Health Care*, the Institute of Medicine (IOM) identified potential sources of racial and ethnic disparities in healthcare and provided recommendations for healthcare professionals, health systems, and policy makers [9,10]. However, racial and ethnic disparities in the United States continue. According to the 2018 National Healthcare Quality and Disparities Report, Black, American Indian or Alaska Native, and Native Hawaiian or Pacific Islander patients continue to receive poorer care than White patients on 40% of the quality measures included, with little to no improvement over the previous decades.

For health systems, the IOM recommendations include developing and implementing interventions that promote the use of evidence-based guidelines, structuring payment systems that ensure equitable access, cultivating communication and trust between providers and patients, employing language interpretation services, and engaging in multidisciplinary treatment and preventative care teams [11]. While many health systems have incorporated these recommendations, the novel coronavirus (COVID-19) pandemic exposed and compounded serious flaws in the American health care system [12].

One healthcare setting largely overlooked by researchers in the deployment of equitable services is the community pharmacy. Pharmacy practice models have highlighted community pharmacies’ unique position in connecting individuals to care [12,13,14]. Yet, little research has been performed in this setting to understand its delivery of equitable care. As one of the most accessible healthcare systems, community-based pharmacies can employ many of the IOM’s recommendations to target and reduce racial and ethnic healthcare disparities. As pharmacy practice shifts its focus toward population health care needs that serve public health, there is a need to understand community-based pharmacies contributions to the reduction in health disparities [15,16]. This scoping review was conducted to identify what community-based pharmacies are doing to target health and healthcare disparities in their areas. In addition to identifying interventions, we compared these interventions to the IOM recommendations for health systems to identify gaps and opportunities for community pharmacies to reduce healthcare disparities. 

## 2. Materials and Methods

A scoping review with a systematized methodology was conducted, as this method offered the researchers an opportunity to report evidence of community pharmacy-based interventions that addressed racial and ethnic health care disparities [17]. Three researchers (TH, FH, JR) evaluated pharmaceutical services in community and ambulatory care pharmacies using PRISMA (Preferred Reporting Items for Systematic Reviews and Meta-Analyses) guidelines [18]. Additionally, each intervention as described in each paper was mapped against the IOM’s recommendations for health systems to reveal remaining evidence gaps that will inform practice innovation in the field and inspire future research [19,20,21]. 

The population for this study was community-based pharmacy. For this study, community-based pharmacy is defined as a setting where pharmacists provide care within traditional community pharmacies, physician offices, ambulatory and outpatient clinics, patient-centered medical homes, and other community-based settings [22]. The interventions studied were pharmaceutical care services whose goal was to reduce health and/or healthcare disparities between racial and ethnic minoritized groups. For the purposes of this study, the following were included as racial and ethnic minoritized groups: African Americans or Black Americans, Hispanic Americans or Latinx Americans, Asian Americans, Native Hawaiian or other Pacific Islander Americans, and American Indians or Alaska Natives.

This review was submitted for registration in PROSPERO (No. CRD42022307405) and followed PRISMA systematic review reporting guidelines [23]. 

### 2.1. Identifying Relevant Studies

The authors searched PubMed for articles using the following combination of MeSH terms, title words, and keyword synonyms, including their multiple variants: healthcare disparity OR health equity OR disparities, health status OR minority health) OR Health Care Quality, Access, and Evaluation AND African Americans OR Indians, North American OR Indigenous Peoples OR Arabs OR Asian Americans OR Hispanic Americans OR Minority Groups AND Pharmacists OR Pharmacy Technicians OR Practice Patterns, Pharmacists OR Pharmacy Team OR Pharmacy OR Pharmaceutical Services OR Pharmacy Services AND 2003:3000/12/12[pdat]. Included studies were those describing pharmaceutical services that were led by a community-based pharmacist or in a community-based pharmacy setting intended to reduce racial/ethnic health and healthcare disparities and met other inclusion criteria including publication date [“1 January 2003”[PDat] “26 March 2021”[PDat]. This date was chosen based on the 2003 release date of the Institute of Medicine’s report “Unequal Treatment: Confronting Racial and Ethnic Disparities in Health Care” that included recommendations for health system interventions to reduce health and healthcare disparities [9]. If a manuscript was published within the search parameters, but the intervention described within the manuscript began before the release of the IOM’s statement, that paper was also not eligible. To supplement the papers obtained in our database searches, we additionally reviewed bibliographies and added previously recognized landmark studies.

### 2.2. Select the (Relevant) Studies

All articles identified from the search were imported into a database displaying the title, PMID, author, and abstract. Two researchers (FH and TH) separately reviewed the article titles and applied the screening tool (inclusion criteria are explained below) to determine their eligibility for full-text review. Each researcher then conducted a full-text review and selected eligible articles. Eligible studies met inclusion criteria: (1) represented an original study; (2) included at least one intervention led by a community-based pharmacist or in a community-based pharmacy setting designed to reduce racial and ethnic health or healthcare disparities; (3) presented data for racial and/or ethnic minority populations in the US; and (4) reported findings in English. We did not include conference abstracts or unpublished studies. Furthermore, articles were excluded for wrong study design if they did not detail a specific intervention led by a pharmacists or their support staff; wrong setting if they did not take place in a community-based pharmacy; and wrong patient population if they did not have majority of the sample population come from at least one minoritized racial or ethnic minoritized groups. Non-US studies and articles unavailable for full-text access were also disregarded. The team reviewed the selected articles, discussed the findings, and resolved disagreements on study selection and data extraction by consensus and through discussion.

### 2.3. Charting the Data

**Figure 1:**
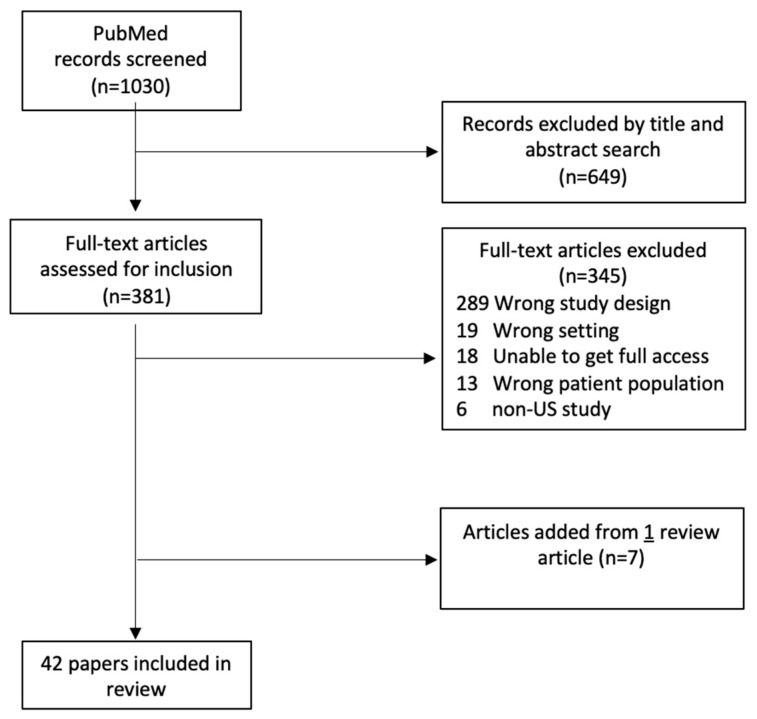
PRISMA flow diagram for Review.

We adopted a descriptive analytical approach to the included articles. Responses were charted in an Excel database. Included articles were compared to the IOM recommendations for health systems (Figure 1). 

### 2.4. Collating, Summarizing, and Reporting the Results

Articles were collated, quantitatively summarized, and thematically analyzed to identify patterns based on title and abstract. Spreadsheets and tables were produced that categorized each theme with a different color. To determine the effect of each type of intervention, we classified studies by intervention type and IOM categorizations. We also grouped studies with similar intervention components together to assess the impact of the combination of interventions because the majority of intervention techniques used more than one intervention [24]. A summary of the IOM recommendations relevant to health-system interventions appears in Table 1.

## 3. Results

The original search yielded 1030 articles, 649 of which were deleted after reviewing the titles and abstracts. An additional 345 articles were discarded after reviewing the articles in their entirety, resulting in 36 articles. In the process of reviewing articles identified through the initial searches, one of these articles was a review article. From this review article, 7 articles were identified through cited references, bringing the total to 42 articles for this review. Table 1 details the articles found, including the study setting, identified healthcare disparity or inequity, a brief description of the intervention, racial/ethnic minority group targeted, and study findings. 

### 3.1. Publication Years and Study Design Characteristics

In total, 4 of the 42 publications were published within five years following the IOM guidelines. Furthermore, 15 articles were published between 2008–2012, which was more than five but less than 10 years since the release of the IOM recommendations, with the remaining articles being published greater than 10 years since the release of the 2003 IOM recommendations. Among the 42 articles, 11 articles used an experimental design, 20 employed an observational design, and 11 used an implementation science design. 

### 3.2. Study Subjects

This review’s studies mostly focused on African American (*n* = 21) and Hispanic (*n* = 23) groups. Interventions were developed for American Indians and Alaskan Natives in 4 of the articles and for Asian individuals in 2 of the articles. The majority of the studies (*n* = 39) focused on the general population. Two studies’ research participants were pediatric patients, and one studies’ research participants were older adults. Majority of the studies targeted patients with diabetes (*n* = 17) and hypertension (*n* = 13). The remaining studies targeted general health literacy and counseling barriers (*n* = 8), vaccinations (*n* = 4), obesity and other weight related parameters (*n* = 4), asthma (*n* = 3), HIV (*n* = 2), smoking cessation (*n* = 2), dyslipidemia (*n* = 2), cancer prevention (*n* = 1), ophthalmic care (*n* = 1), and psychiatric illness (*n* = 1). 

### 3.3. Pharmaceutical Care Services and Community-Based Setting

In the articles reviewed, pharmaceutical care services included medication and/or disease-state management (*n* = 17), patient counseling and education (*n* = 7), communication services, including language assistance (*n* = 8) and health literacy (*n* = 2), point of care testing (*n* = 5), and telemedicine (*n* = 1). Most of the pharmaceutical care services were rendered in the traditional community pharmacy (*n* = 13) and community-based clinic (*n* = 13) setting. The remaining services were rendered in community outreach sites (*n* = 9), safety-net or federally qualified health systems (*n* = 4), patient homes or nursing homes (*n* = 2), and community-based health systems (*n* = 1). Additional information regarding the articles reviewed can be found in Table 2. 

### 3.4. IOM Recommendations and Gap Analysis 

The IOM recommendations (IOMR) most frequently addressed by the included studies were: **IOMR 5–11**, implement multidisciplinary treatment and preventive teams (*n* = 30); **IOMR 5–12**, implement patient education programs to increase patients’ knowledge of how to best access care and participate in treatment decisions (*n* = 13); **IOMR 5–9**, support the use of interpretation services where community need exists (*n* = 12); **IOMR 5–10**, support the use of community health workers (*n* = 10); **IOMR 5–6**, promote the consistency and equity of care through the use of evidence-based guidelines (*n* = 9); **IOMR 5–7**, structure payment systems to ensure an adequate supply of services to minority patients, and limit provider incentives that may promote disparities (*n* = 8), addressed by less than a third of the included studies; **IOMR 5–8**, Enhance patient-provided communication and trust by providing financial incentives for practices that reduce barriers and encourage evidence-based practice, only addressed by one study. Evidence of the recommendations carried out by the studies are revealed in Table 3.

## 4. Discussion

In this scoping review, we assessed the provision of pharmaceutical care services targeted for racial and ethnic minoritized populations in community-based pharmacies. To our knowledge, this is the first study to assess whether community-based pharmacies have carried out the recommendations as laid out by the IOM in their paper *Unequal Treatment: Confronting Racial and Ethnic Disparities in Health Care*. This research found that community-based pharmacy services address racial and ethnic inequities in a variety of ways, including the types of interventions employed, as well as the ethnicities and conditions of the sample populations. This paper also highlights the important role of community-based pharmacies in carrying out the IOM’s recommendations and reveals some areas for research and progress. 

Having pharmacists on the multidisciplinary healthcare team has long been established as being beneficial for patients in a variety of clinical settings [25,26,27]. As a result, it came as no surprise that our study showed that community-based pharmacies and pharmacists excel in carrying out **IOMR 5–11:** *Implement multidisciplinary treatment and preventive team.* Using medication therapy management (MTM) programs and collaborative practice agreements (CPA) with physicians, pharmacists have improved health measures such as blood pressure, blood glucose, and cholesterol levels [28,29]. Thus, it is not surprising that including pharmacists on the healthcare team in areas that provide services to historically marginalized and minoritized patients also demonstrate improved health measures. Similarly, pharmacists seamlessly integrate into preventative care teams. The ability to do so is made possible by the fact that pharmacists are frequently regarded as trustworthy members of their communities, which opens the door to offering preventive screenings. From screenings for chronic conditions such as hypertension and diabetes to undiagnosed HIV infections and diabetic retinopathy, the accessibility of community-based pharmacists creates a natural touch point for health screenings for patients who would otherwise not present to a physician’s office. Our results also reveal that pharmacists are also providing screening services beyond the four walls of the pharmacy at after-school programs [30], college campuses [31], and health fairs [32]. As community pharmacy continues to expand services offered, preventive care appears to be an accessible way pharmacists can work to reduce health disparities in their communities.

Our data also revealed that community-based pharmaceutical services implemented IOM recommendations **5–12, 5–9, 5–10, 5–6, and 5–7**, though to a lesser extent than **IOMR 5–11**. **IOMR 5–9** is perhaps one of the most important recommendations that community-based pharmaceutical services should carry out, as there is little question concerning that misinterpreting medical instructions can be fatal. Research indicates that community pharmacists do not regularly or effectively use language-access services in daily practice [33,34,35,36,37,38]. Yet, with more than 300 languages spoken or signed in the US and projections that the US will be the largest Spanish-speaking country by 2050, [39,40] access to pharmaceutical services that provide effective communication between patients and pharmacists is more crucial than ever. Community-based pharmacies must be committed to working with their communities to develop culturally and linguistically appropriate pharmaceutical care that addresses disparities in racial and ethnic minority populations. 

For **IOMR 5–12**, the evidence supports that community-based patient education programs that allow underserved minorities to be a part of their own treatment eventually lead to improvements in the population health of such groups [41]. Many of these studies resulted in overall improvements for BIPOC patients with diabetes [42,43,44]. According to our findings, the use of **IOMR 5–10** for community-based pharmaceutical services is still relatively new, but it has the potential to enhance medication adherence and health outcomes in patients who have limited access to healthcare. Community health workers (CHW) are lay community members who share similar socioeconomic positions, racial or ethnic identities, and linguistic experiences to the patients they serve [45]. While there are limited studies evaluating the value of CHW and community-based pharmacist collaborations, the effectiveness of CHW and pharmacist-provided care independently have been demonstrated in the literature and may be dependent on the role of the CHW and the needs of the patient population [46,47,48,49]. 

While this study highlights the many contributions community-based pharmaceutical services make in reducing racial and ethnic healthcare disparities, gaps in recommendations did appear. Though carried out, more work is needed in accomplishing **IOMR 5–6:** *Promote the consistency and equity of care through the use of evidence-based guidelines*. The employment of evidence-based strategies has long been essential in the provision of pharmaceutical care [50,51,52]. Studies have found that pharmacists face barriers as a result of limited access to resources related to evidence based medicine and patient overload [52,53]. Furthermore, gaps in care for other vulnerable populations have been identified using national consensus guidelines [54]. This suggests that more needs to be performed to ensure that evidence-based practices are promoted throughout pharmacy practice, but especially for vulnerable groups such as racial and ethnic minorities who continue to experience substandard outcomes in the delivery of care received. In their statement on racial and ethnic disparities in health care, the American Society of Health-System Pharmacists encourages pharmacists to use evidence-based guidelines for patient care and to confront the cultural divide that exists between the demands of their profession and the deeply held beliefs of their patients. Further stating that this will provide the advantages of “consistency, predictability, and objectivity”, particularly in cases where there is evidence of various outcomes or reactions among racial and ethnic minoritized groups. Additionally, while this statement came out in 2008 and the IOM recommendations in 2003, our data continues to suggest that more work is needed to promote this recommendation throughout community-based pharmacy practice. 

In this scoping review, only one study focused on **IOMR 5–8**: Enhanced patient-provider communication and trust by providing financial incentives for practices that reduce barriers and encourage evidence-based practice. This is not surprising since community-based reimbursement models are traditionally attributed to the prescription product rather than to patient education. There are numerous challenges in the current pharmacy reimbursement system that does not allow for incentives for patient education [55]. Innovative models of care are being piloted to change the system so that it empowers the profession of pharmacy to improve lives for patients across the country [56]. Emerging research that investigates these novel community pharmacy practice models provides real-world examples of these models and discusses reimbursement and sustainability pathways is a step in the right direction to challenge the current system and ensure that barriers to care for patients in greatest need are reduced for community-based pharmacists.

## 5. Conclusions

This study highlights the many contributions community-based pharmaceutical services provide in reducing racial and ethnic healthcare disparities. Community-based pharmacies employ many of the Institute of Medicines’ recommendations to target and reduce racial and ethnic healthcare disparities, but more progress may be needed. Future work should focus on ensuring that the IOM’s recommendations are carried out throughout pharmacy practice and are accessible to all racial and ethnic minoritized populations. 

## Figures and Tables

**Table 1:** IOM Recommendations Relevant to Health-System Interventions [12].

Recommendation	Summary
5–6	Promote the consistency and equity of care through the use of evidence-based guidelines.
5–7	Structure payment systems to ensure an adequate supply of services to minority patients and limit provider incentives that may promote disparities.
5–8	Enhance patient-provided communication and trust by providing financial incentives for practices that reduce barriers and encourage evidence-based practice.
5–9	Support the use of interpretation services where community need exists.
5–10	Support the use of community health workers.
5–11	Implement multidisciplinary treatment and preventive care teams.
5–12	Implement patient education programs to increase patients’ knowledge of how to best access care and participate in treatment decisions

**Table 2:** Characteristics of community-based pharmaceutical services that target racial/ethnic health care disparities.

Author, Year	Target Population *	Targeted Intervention	Pharmaceutical Service Type
Anderegg MD, Gums TH, et al., 2016	African American Hispanic	MTM visits	Medication Optimization
Blake SC, McMorris K, et al., 2010	African American Caucasian **	Health communication training for pharmacists	Language/Literacy
Caballero J, Souffrant G, et al., 2008	Hispanic	Pharmacists provide psychiatric services	Medication Optimization
Carrillo M, Sias J, et al., 2018	Hispanic, Spanish speakers	Expanded and enhanced diabetes health education by an interprofessional team	Patient Education or Counseling
Carter BL, Coffey CS, et al., 2015	African American Hispanic	MTM visits	Medication Optimization
Collins B, Bronson H, et al., 2018	Non-Hispanic Black Non-Hispanic White ** Hispanic of any race	HIV testing program	Point of Care Testing
Congdon, H.B., Dowling, et al., 2013	Hispanic	MTM visits	Medication Optimization
Elliott JP, Harrison C, et al., 2015	Black, Non-Hispanic White, Non-Hispanic	Pharmacist and student pharmacist directed pediatric health screenings	Point of Care Testing
Enfinger F, Campbell K, et al., 2009	African Americans Caucasians ** Hispanic	Pharmacy team consultations during regular physician visits	Medication Optimization
Gazmararian J, Jacobson KL, et al., 2010	African American White **	Health communication training for pharmacists	Language/Literacy
Geiger R, Steinert J, et al., 2018	American Indian/Alaska Natives	Pharmacist conducted chart reviews of HCV patients	Medication Optimization
Gerber BS, Cano AI, et al., 2010	Latino (Hispanic)	Use of bilingual health promoter	Language/Literacy
Isaacs D, Riley AC, et al., 2017	African American	Interdisciplinary health fairs and community health outreach events	Point of Care Testing Patient Education or Counseling Immunizations
Jameson JP and Baty JP, 2010	Non-white	Disease control by a pharmacist	Medication Optimization
Kirwin JL, Cunningham RJ, et al., 2010	White ** Black Hispanic Asian Unknown	Pharmacist delivered, primary care, physician-focused intervention	Medication Optimization
Lam AY and Chung Y, 2008	Multiethnic Asian	Pharmacist-conducted on-site influenza vaccination service	Immunizations
Lee J, McKennett M, et al., 2019	Hispanic or Latino	Interdisciplinary community health fair	Immunizations
Martin SL, Williams E, et al., 2015	American Indians/Alaskan Natives	Interprofessional diabetes clinic	Medication Optimization
Matthews PH, Darbisi C, et al., 2009.	Latino	Touch-screen information kiosks with online access as a tool for providing culturally and linguistically relevant diabetic information	Language/Literacy
Meyerson BE, Carter G, et al., 2016	African American	Voucher outreach program for HIV home testing at three pharmacies	Point of Care Testing
Moreno G, Tarn DM, et al., 2009.	Latino	Access to non-English language pharmacy service	Language/Literacy
Muzyk, AJ., Muzyk, TL, et al., 2004.	Hispanic African American Asian or Pacific Islander	Language-assistance services	Language/Literacy
Navarrete, JP, Padilla, ME, et al., 2014	Hispanic	Pharmacist-operated HPV vaccine program	Immunizations
Ndefo, UA, Davis, PN, et al., 2019	African American	Home visits by pharmacists	Medication Optimization
Odegard PS, Lam A, et al., 2004	Asian, whose native language was not English	Oral and written asthma education	Language/Literacy
Owsley C, McGwin G Jr, et al., 2015	African American Hispanic Haitian Cuban American	Telemedicine screening	Telemedicine
Oyetayo OO, James C, et al., 2011	Hispanic	Diabetes counseling, education, and monitoring every 3 months by pharmacists	Patient Education or Counseling
Rick R, Hoye RE, et al., 2017	American Indian	Motivational interviewing and diabetes self-management education	Patient Education or Counseling
Sansgiry, SS., Chanda S, et al., 2007.	Hispanic	Non-English language pharmacy service	Language/Literacy
Sharif I, Lo S, 2006	Spanish-speakers	Non-English language pharmacy service	Language/Literacy
Sharp LK; Tilton JJ, et al., 2018	African American Hispanic	CHW support in addition to clinical pharmacist support	Medication Optimization
Shireman TI, Svarstad BL, 2016.	Black	Novel tools for improving adherence	Medication Optimization
Shiyanbola OO; Kaiser BL, et al., 2021	Black/African American	Pharmacist-led educational program on diabetes	Patient Education or Counseling
Sisson EM; Dixon DL; et al., 2016	African American White ** Asian Hispanic Other	Pharmacists provided medication management.	Medication Optimization
Snella KA; Canales AE; et al., 2006	African American Hispanic	Pharmacist-led diabetes, HTN, and dyslipidemia screening	Point of Care Testing
Soller RW, Chan, P, et al., 2012	Spanish-speaking	MTM provided by pharmacists	Language/Literacy Medication Optimization
Svarstad, BL, Kotchen, JM, et al., 2013	Black	Novel tools for improving adherence	Medication Optimization
Tao LS; Hart P; et al., 2003	African American Hispanic/Latino	Pharmacist led education	Patient Education or Counseling
Valencia, V, Padilla, ME, 2015.	Hispanic	Education sessions led by community pharmacists	Patient Education or Counseling
Victor RG; Blyler CA, 2018	Non-Hispanic Black	Barbers promoted follow-up with pharmacists	Medication Optimization
Victor RG; Lynch K, et al., 2018	Black	Barbers promoted follow-up with pharmacists	Medication Optimization
Wheat L; Roane TE, et al.,	Black Native American Caucasian	Collaboration between pharmacists and CHWs in identifying and addressing barriers to medication adherence and improving health outcomes for patients diagnosed with hypertension with or without diabetes	Medication Optimization

* Race and ethnicity defined as appeared in the reference; ** Non-minoritized patients were included for comparison and/or not excluded though not the target population outlined in the study’s objective.

**Table 3:** Evidence gaps in community-based pharmaceutical services and IOM’s recommendations.

	Institute of Medicine Recommendations on Health Disparities						
	Health System Interventions						Patient Education and Empowerment
	**5–6**	**5–7**	**5–8**	**5–9**	**5–10**	**5–11**	**5–12**
Anderegg et al., 2016							
Blake SC, et al., 2010							
Caballero J, 2008							
Carrillo et al., 2018							
Carter et al., 2015							
Collins et al., 2018							
Congdon et al., 2013							
Elliott et al., 2015							
Enfinger et al., 2009							
Gazmararian J, 2010							
Geiger et al., 2018							
Gerber et al., 2010							
Isaacs et al., 2017							
Jameson JP and Baty JP, 2010							
Kirwin JL, 2010							
Lam et al., 2008							
Lee et al., 2019							
Martin et al., 2015							
Matthews et al., 2009							
Meyerson et al., 2016							
Moreno et al., 2009							
Muzyk et al., 2004							
Navarrete et al., 2014							
Ndefo et al., 2019							
Odegard PS, et al., 2004							
Owsley et al., 2015							
Oyetayo et al., 2011							
Rick et al., 2017							
Sansgiry et al., 2007							
Sharif et al., 2006							
Sharp et al., 2018							
Shireman & Svarstad, 2016							
Shiyanbola et al., 2021							
Sisson et al., 2016							
Snella et al., 2006							
Soller et al., 2012							
Svarstad et al., 2013							
Tao et al., 2003							
Valencia et al., 2015							
Victor et al., 2018							
Victor et al., 2019							
Wheat et al., 2020							

## Data Availability

Data supporting this study are included within the article.
